# Antioxidative Stress Metabolic Pathways in Moderately Active Individuals

**DOI:** 10.3390/metabo13090973

**Published:** 2023-08-27

**Authors:** Shamma Almuraikhy, Najeha Anwardeen, Asmma Doudin, Maha Sellami, Alexander Domling, Abdelali Agouni, Asmaa A. Althani, Mohamed A. Elrayess

**Affiliations:** 1 Biomedical Research Center, Qatar University, Doha P.O. Box 2713, Qatar; 2 Groningen Research Institute of Pharmacy, Drug Design, Groningen University, 9713 AV Groningen, The Netherlands; 3 Physical Education Department (PE), College of Education, Qatar University, Doha P.O. Box 2713, Qatar; 4 College of Pharmacy, QU Health, Qatar University, Doha P.O. Box 2713, Qatar; 5 Department of Biomedical Sciences, College of Health Science, QU Health, Qatar University, Doha P.O. Box 2713, Qatar

**Keywords:** physical activity, oxidative stress, metabolomics, glutathione, plasmalogen, phosphatidylcholine, vitamin A, xanthine

## Abstract

Physical activity (PA) is known to have beneficial effects on health, primarily through its antioxidative stress properties. However, the specific metabolic pathways that underlie these effects are not fully understood. This study aimed to investigate the metabolic pathways that are involved in the protective effects of moderate PA in non-obese and healthy individuals. Data on 305 young, non-obese participants were obtained from the Qatar Biobank. The participants were classified as active or sedentary based on their self-reported PA levels. Plasma metabolomics data were collected and analyzed to identify differences in metabolic pathways between the two groups. The results showed that active participants had increased activation of antioxidative, stress-related pathways, including lysoplasmalogen, plasmalogen, phosphatidylcholine, vitamin A, and glutathione. Additionally, there were significant associations between glutathione metabolites and certain clinical traits, including bilirubin, uric acid, hemoglobin, and iron. This study provides new insights into the metabolic pathways that are involved in the protective effects of moderate PA in non-obese and healthy individuals. The findings may have implications for the development of new therapeutic strategies that target these pathways.

## 1. Introduction

Physical activity (PA) has been shown to lower body fat percentage and improve blood pressure and lipids profile, thus reducing the risk of cardiovascular diseases [1]. Recent joint guidelines of the American College of Sports Medicine and the American Heart Association have recommended 150 min of moderate activity (30 min, 5 days/week) or 60 min of vigorous PA (20 min on 3 days) for all adults. Particularly, walking regularly can improve sugar metabolism and cardiorespiratory fitness, leading to better outcomes and quality of life [2]. Regular exercise in non-obese individuals was shown to improve glucose tolerance and insulin sensitivity, as well as various lipid parameters, blood pressure, and fibrinolytic activity [3,4,5]. Even exercise programs that produced little or no change in maximal oxygen uptake (VO_2_ max), such as long-term walking, reported improvement in insulin sensitivity [6,7].

Physical activity can induce long-term adaptations in cellular metabolism and immune cells, including decreased oxidative stress status with a concomitant rise in antioxidant factors [8]. Analyzing the functional interactions between physical exercise and the immune system is extremely complex due to the multiplicity of the underlying mediators. These include the type, frequency, and intensity of the exercise, as well as individual variability of the immune system in response to exercise-induced stress [9].

Oxidative stress refers to the disparity between the generation of free radicals and an organism’s capacity to counteract them using antioxidants [10]. Free radicals are molecules with high reactivity, possessing one or more unpaired electrons that render them unstable. These radicals are produced during routine metabolic processes, both at rest and during exercise.

The antioxidant response following physical exercise in human plasma can be evaluated using protein oxidation, lipid oxidation, and exogenous antioxidant utilization, including total antioxidant capacity (TAC). Other examples include antioxidant enzymes such as superoxide dismutase (SOD) and glutathione peroxidase (GPX), which are elevated during physical training [11]. Additionally, increased water and lipid soluble endogenous antioxidant levels, such as ascorbic acid and alpha tocopherol, can be considered [12]. Similarly, proteins that are associated with oxidative stress tend to increase with a steady-state exercise [13].

The metabolomics of human serum samples can provide a snapshot of the current metabolic state of an individual. The levels of circulating metabolites are influenced by both genetic and environmental factors, and can further depend on age, sex, menopause, and diet of the study participants. Previous investigations primarily focused on metabolomics using urine and blood, revealing significant concentration changes in 196 metabolites following aerobic or resistance exercise [13]. However, as highlighted by Schranner et al. [13], “metabolite concentrations change in different directions after exercise.” Considering that earlier studies examined metabolites in diverse athlete categories and during the acute exercise phase, it became imperative to scrutinize their fluctuations in active, healthy individuals and throughout extended adaptation periods. This approach aims to offer a richer dataset to practitioners and clinicians, enhancing the depth of information available.

A better understanding of these relationships is pivotal for the planning of metabolomics studies involving human subjects and the interpretation of their results [14]. Metabolomics offers a quantitative measurement of the metabolic profiles associated with exercise in moderately active non-obese participants to identify biomarkers associated with moderate activity. Non-targeted metabolomics allows the detection of changes in response to various physiological states such as pre-/post-exercise, and enables the identification of metabolic signatures with potential translational impact for the public [15].

The emerging data highlight the involvement of antioxidative stress pathways with overall protective phenotypes. Therefore, the objective of this study is to identify the metabolic pathways underlying the protective effect of moderate PA in non-obese, apparently healthy individuals using an untargeted metabolomics approach.

## 2. Materials and Methods

### 2.1. Data Source and Study Participants

Data were collected from 305 participants from the Qatar Biobank, including questionnaires related to physical activity and laboratory results for 66 clinically-relevant metabolic traits, such as measurements of systolic and diastolic blood pressure, waist to hip ratio (WHR), body mass index (BMI), clinical chemistry, and endocrinology tests (Table 1). In addition, metabolomics data were collected on over 1000 metabolites. The study was approved by the Institutional Review Boards of the Qatar Biobank (QF-QBB-RES-ACC-00066) and Qatar University (QU-IRB 1716-E/22). All participants provided informed consent. Physically active participants were identified as those who practice any type of moderate-intensity physical activity (i.e., walking, jogging, swimming, or riding) for at least 150 min per week (moderate activity) and not more than 300 min/week according to the ACSM, WHO, and CDC guidelines and the Physical Activity Guidelines for Americans. Inclusion criteria included individuals who were young (20–30 years old), lean/overweight (BMI: 20–30 kg/m^2^), and healthy (no chronic diseases such as diabetes, glaucoma, macular degeneration, blood clots, cardiovascular disease, bariatric surgery, or cancer). Accordingly, among all participants, 42% were physically active and 58% were sedentary.

### 2.2. Metabolomics

Established protocols were used for untargeted metabolomics of serum samples from all participants using the Metabolon platform [15]. To measure the metabolites, we used a Waters ACQUITY ultra-performance liquid chromatography (UPLC) (Waters Corporation, Milford, MA, USA) and a Thermo Scientific Q-Exactive high resolution/accurate mass spectrometer interfaced with a heated electrospray ionization (HESI-II) source and Orbitrap mass analyzer operated at 35,000 mass resolution (Thermo Fisher Scientific, Waltham, MA, USA). A detailed description of the methodology was previously provided [15]. To identify compounds, hits were compared with existing library entries of purified standards of over 3300 purified standard compounds. Compounds were then assigned to various categories according to their sources, as previously described [16].

### 2.3. Statistical Analysis

The metabolomics data were log-transformed. Multivariate analysis, including unsupervised principle component analysis (PCA) and supervised orthogonal partial least square-discriminant analysis (OPLS-DA), were run using SIMCA^®^ (version 16.0.1) software (Sartorius Stedim Data Analytics AB, Sweden). Two outliers were removed after PCA analysis as part of quality control prior to OPLS-DA. R version 4.0.3 (Vienna, Austria) was used to perform linear models for each metabolite (as the response variable) versus physical activity (active/sedentary) (as the explanatory variables). The model also included the following confounders: age, gender, BMI, and principle components 1 and 2 from the PCA analysis (PC1 and PC2 explain the dominant and secondary source of variability in the dataset, respectively, provided PC2 is uncorrelated with PC1. This method is applied to complex datasets to identify strong patterns and/or additional nuances present in the data). Correcting for these components accounts for their influence and focuses on the effect of the predictor variable on the dependent variable. The multiple testing correction method (False Discovery Rate (FDR)) was used to adjust the nominal *p*-values. FDR < 0.1 was considered statistically significant. Functional enrichment analysis was run on all *p*-value ordered metabolite lists from linear models performed in the study. This analysis was conducted based on Fisher’s exact test, which was followed by the FDR multiple testing correction method. The sub-pathways were previously predefined using Metabolon, and those with less than three top hits were dropped.

## 3. Results

### 3.1. Comparing the Effect of Physical Activity on Clinical Metabolic Traits

When assessing the impact of physical activity, the active participants showed lower HOMA-IR, average pulse rate, mean cell hemoglobin concentration, GGT, and GGT 2 than their sedentary counterparts (*p*-value < 0.05). Additionally, the active participants exhibited higher level of handgrip (left and right), maximum heart rate, creatine kinase, AST, and HDL cholesterol (*p*-value <0.05) (Table 1). When conducting these comparisons in males and females separately, data suggested lower levels of insulin, HOMA-IR, C-peptide, GGT, GGT2, and triglycerides in active compared with sedentary males, but not in females. Data also revealed that active males, but not females, had higher level of handgrip (right and left). Active females, but not males, had lower maximum heartbeat and % of basophils, but higher % of lymphocytes, HDL, and total protein compared with their sedentary counterparts (Table A1).

### 3.2. Multivariate Analysis of Metabolites Differentiating between Active and Sedentary Individuals

**Figure 1:**
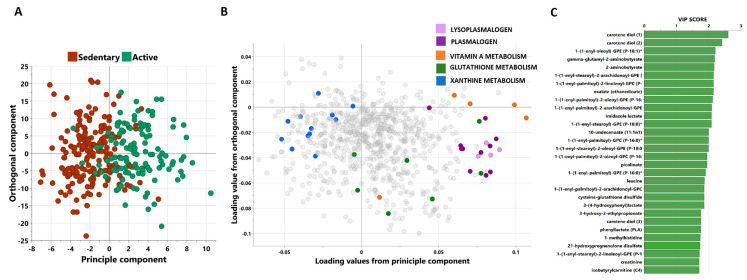
A score plot from the OPLS-DA model comparing active vs sedentary participants showing the class-discriminatory component 1 (x-axis) versus l Orthogonal component 1 (y-axis) (**A**), the corresponding loadings plot from the OPLS-DA model showing metabolites that belong to significantly enriched pathways (**B**), and a variable importance in projection (VIP) plot for the OPLS-DA model as a quantitative estimation of the discriminatory power of the top 30 metabolites between active and sedentary participants (**C**). Asterisks (*) indicated on IDs of some metabolites refer to compounds that have not been officially confirmed based on a standard, but their identities are known with confidence.

Non-targeted metabolomics was applied to determine the metabolic signatures of the 305 subjects. Two subjects deemed outliers from unsupervised principle component analysis (PCA) were removed from further analysis. OPLS-DA was performed to identify components that best differentiated between the active and sedentary cohorts. The model identified one predictive and two orthogonal components from the data, capturing 14.8% variance in the data and 52.3% variance between the study groups. The score plot (Figure 1A) shows a clear distinction between sedentary (red) and active (green) participants, whereas the loading plot (Figure 1B) shows metabolites responsible for differentiating the two groups. These include xanthine metabolites enriched among the sedentary group, and lysoplasmalogen, plasmalogen, vitamin A, and glutathione metabolites enriched among the active participants. A variable importance in projection (VIP) plot for OPLS-DA model (Figure 1C) shows a quantitative estimation of the discriminatory power of the top 30 metabolites between active and sedentary participants.

### 3.3. Univariate Analysis of Metabolites Differentiating between Moderately Active and Sedentary Individuals

**Figure 2:**
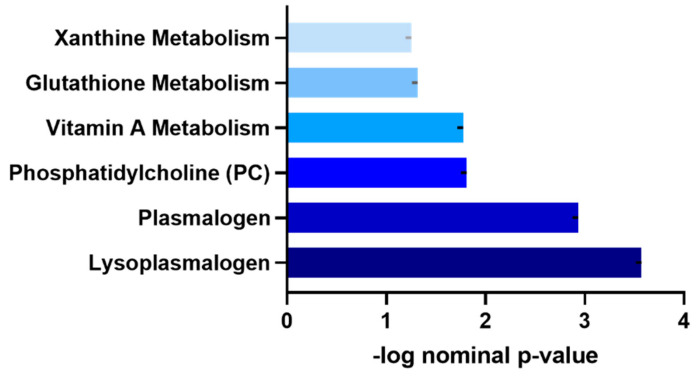
Bar plot of functional enrichment analysis results of active vs sedentary status based on metabolite ranks by *p*-value using Fisher’s exact test.

A linear model was used to assess the significance of metabolite associations with physical activity status after correcting for gender, age, BMI, and principal components. Data indicated 14 metabolites associated with activity at (FDR ≤ 0.1) (Table 2). The univariate analysis confirmed results identified through the OPLS-DA multivariate, including those belonging to vitamin A and xanthine metabolism (Figure 2).

### 3.4. Functional Enrichment Analysis of Metabolites Differentiating Moderately Active and Sedentary Non-Obese Individuals

Functional enrichment analysis of metabolites was performed using metabolite ranks by *p*-value from the univariate analysis. Pathway enrichment analysis revealed an over-representation of lysoplasmalogen, plasmalogen, phosphatidylcholine (PC), vitamin A, glutathione, and xanthine metabolism (Figure 2), similarly to OPLS-DA (Figure 1B).

### 3.5. Identifying Oxidative Stress-Related Metabolites That Significantly Changed between Sedentary and Active Participants

**Figure 3:**
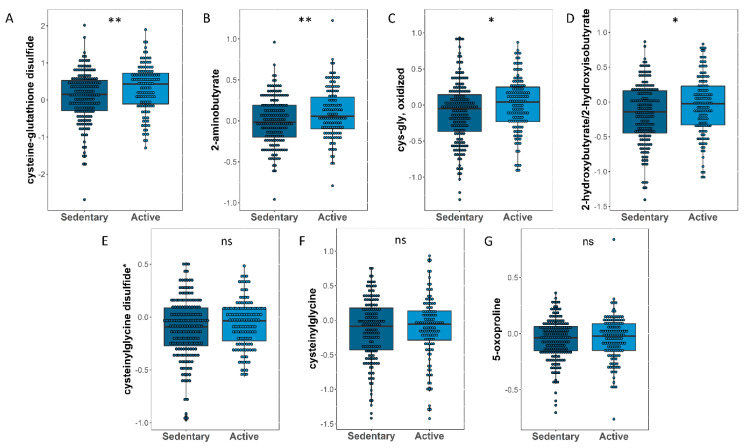
Box plot of metabolites related to glutathione metabolism differentiating between sedentary and active participants, including cysteine-glutathone disulfide (**A**), 2-aminobutyrate (**B**), cys-gly, oxidized (**C**), 2-hydroxybutyrate/2-hydroxyisobutyrate (**D**), cysteinylglycine disulfide (**E**), Cysteinylglycine (**F**) and 5-oxoproline (**G**). Asterisks (*) indicated on IDs of some metabolites refer to compounds that have not been officially confirmed based on a standard, but their identities are known with confidence. **/*/ns in the figure represents *p*-value <0.01/<0.05/not significant respectively.

As glutathione metabolism was one of the enriched pathways associated with moderate physical activity, we sought further characterization of the nature of associated metabolites after correcting for age, gender, BMI, and principal components. The results showed cysteine-glutathione disulfide (Figure 3A), 2-aminobutyrate (Figure 3B), cys-gly oxidized (Figure 3C), and 2-hydroxybutyrate (Figure 3D) with nominal significant elevation in active subjects (*p*-values = 0.003, 0.008, 0.019, and 0.05, respectively), as shown in Table 3 and Figure 3.

### 3.6. Association between Antioxidant Response and Clinical Traits in Active Individuals

**Figure 4:**
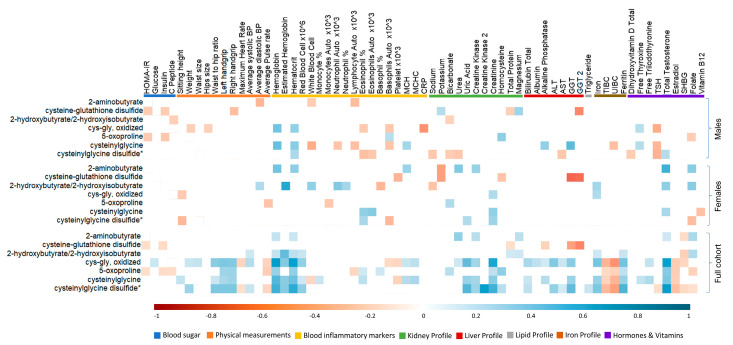
Correlation matrix showing the significant (*p* < 0.05) positive (blue) and negative (red) correlations between glutathione metabolites and clinical markers, and Spearman’s correlations between clinical markers and glutathione metabolites. Asterisks (*) indicated on IDs of some metabolites refer to compounds that have not been officially confirmed based on a standard, but their identities are known with confidence.

In order to understand the implications of elevated oxidative stress-related metabolites on the general health of active individuals, Spearman’s correlation was performed between glutathione metabolites and conventional clinical traits (Figure 4). The data indicated that blood sugar-related traits showed negative correlations with glutathione metabolites, evident mainly in males but not in females. Among physical tests, handgrip was positively correlated with glutathione metabolites, but the average pulse and maximum heart rate were negatively correlated with glutathione metabolites. These associations were not significant when considering each gender separately. Among blood inflammatory markers, hemoglobin and hematocrit were positively correlated with glutathione metabolites in both genders, whereas basophils and platelets were negatively correlated. Liver function enzymes were negatively correlated with oxidative stress markers, except for cysteine-glutathione disulfide, which was positively correlated with multiple liver function enzymes in both males and females. Among the iron profile traits, iron and ferritin were positively correlated with oxidative stress markers, whereas TIBC and UIBC were negatively correlated in total, but not when considering each gender separately. Finally, among the vitamins and hormones, total testosterone was positively correlated with oxidative stress-related metabolites, evident mostly in females but not males. Conversely, estradiol was negatively correlated in general, but not when each gender was considered separately.

## 4. Discussion

Moderate physical activity is associated with improvement in general health through multiple mechanisms, including the activation of antioxidative stress pathways. The metabolic pathways underlying this process in non-obese, apparently healthy individuals are not well characterized. In particular, the metabolic pathways of a moderately active cohort could shed light on the molecular mechanisms with functional relevance or those that can be used as biomarkers of exercise-associated physiological changes.

During moderate-intensity physical exercise, the body generates free radicals as a byproduct of the increased metabolism and oxygen consumption. The body activates several physiological mechanisms to scavenge and neutralize these free radicals. These include the activation of antioxidant enzymes, such as superoxide dismutase (SOD), catalase, and glutathione peroxidase, which neutralize free radicals by breaking them down into less harmful compounds. Additionally, the body increases the production of antioxidant molecules, such as vitamin C and vitamin E, which can neutralize free radicals directly. The production of heat shock proteins, which help to protect cells from damage by free radicals, is also activated. Furthermore, the body activates the Nrf2, a transcription factor that regulates the expression of antioxidant enzymes and antioxidant molecules. Lastly, blood flow to the working muscles increases, helping them to remove waste products, including free radicals, from the body. These mechanisms work together to help protect the body from the potentially harmful effects of free radicals generated during physical exercise [17,18,19,20].

Previous studies have indicated that different biomarkers can be used to assess changes in oxidative stress status with physical activity. Changes in these biomarkers can indicate different outcomes, based on their specific functions [21]. For example, in response to moderate training, lymphocyte glutathione peroxidase and catalase activities increase, while superoxide dismutase activity remains stable [21]. In addition, the type and level of training and individual nutritional status can further impact the metabolic profiles of these biomarkers [21,22]. Several studies have showed that extended exercise has an effect on oxidative stress status in an intensity-dependent manner through the activation of the antioxidant pathway [23]. For example, oxidative stress was reduced after high-intensity exercise compared with low-intensity exercise in rats [24]. Similarity, high-intensity exercise for 4–12 weeks showed a reduction in oxidative stress in both athletes and non-athletes [25]. Studies showed that after four weeks of intensive exercise, levels of thiobarbituric acid reactive substances increased, and total antioxidant status decreased in active and sedentary individuals [26]. Hence, moderate training can be considered a strategy for promoting changes in the balance between pro- and antioxidant products [27]. Another study of sedentary non-obese, overweight, and obese volunteers revealed that a 20-min run at ~70% of VO_2_ max triggers elevated levels of glutathione reductase activity in obese subjects but decreased MCP-1 in non-obese individuals, in addition to decreased EGF in all groups. These findings suggest that glutathione reductase is involved differently in the adaptive metabolic changes and redox responses induced by physical exercise in non-active subjects with different ranges of BMI. Identification of these biomarkers could help to identify people who are at higher risk of developing disease related to oxidative stress [28].

In this study, untargeted metabolomics analysis was utilized to characterize the unique serum metabolic signature of moderately active non-obese participants. The emerging data revealed significant differences in metabolite levels between sedentary and active young non-obese individuals. These differences included a clear signature of oxidative stress-related metabolites that significantly changed between sedentary and active participants.

Our results showed that plasmalogen and lysoplasmalogen metabolism was enriched among the active participants. Plasminogens are a class of membrane glycerophospholipids with unique properties. They contain a vinyl–ether-linked alkyl chain at the sn-1 position of the glycerol backbone and typically, a polyunsaturated fatty acyl chain at the sn-2 position [29,30]. Plasmalogens are critical for human health and have established roles in neuronal development, immune response, and as endogenous antioxidants that protect cells from oxidative stress. Their vinyl–ether bond could indeed be among the first targets for newly formed radicals [29,31,32]. However, the mechanistic bases of these and other biological functions of plasmalogens are not well defined. In contrast, lysoplasmalogens increase membrane fluidity to facilitate cell fusion and migration of circulating inflammatory cells through the endothelium. The lysoplasmalogen may be converted back to plasmalogen in a transacylation reaction. Alternatively, it may be degraded enzymatically by hydrolytic enzymes that include a phospholipase C, a phospholipase D, and lysoplasmalogenase. As the enzyme that cleaves the vinyl–ether bond, lysoplasmalogenase is probably an important enzyme in maintaining the balance between plasmalogen and lysoplasmalogen, thereby preserving membrane stability and function. If levels of lysoplasmalogen rise, they may disrupt and lyse cell membranes. Conversely, if lysoplasmalogen levels are too low, the transacylation reaction cannot occur. Consequently, membrane plasmalogen levels will decrease, thereby disturbing membrane structure and function by that route [33].

Our emerging data revealed that moderate exercise can trigger upregulation of both plasmalogen and lysoplasmalogen levels, highlighting the potential of these metabolites as protection mechanism against oxidative stress.

Furthermore, our results revealed the effect of exercise on increasing vitamin A in the active group. Retinoids (vitamin A) are required for maintaining many essential physiological processes in the body, including normal growth and development as well as healthy vision, immune system, reproductive functions, and skin. Additionally, vitamin A possesses an antioxidative stress function conferred by the hydrophobic chain of polyene units that can quench singlet oxygen, neutralize thiyl radicals, and stabilize peroxyl radicals. Because of its structures, vitamin A can autoxidize when O_2_ tension increases, thereby constituting the most effective antioxidant at low-oxygen tensions, which are typical of physiological levels found in tissues [34]. In this study, we confirmed the enrichment of vitamin A in active individuals, providing evidence of an additional antioxidant mechanism associated with moderate physical activity.

Our results also showed that PC were enriched among the active participants. PC, the most abundant of the phospholipids, have several metabolic functions in organs such as the liver and the intestine, as well as important structural and signaling functions in biological membranes [35,36]. PC liposomes have both neuroprotective and antioxidative properties through the inhibition of microglial activation [37]. PC decreased oxidative stress in the sciatic nerve by increasing antioxidant levels (glutathione, glutathione peroxidase, and superoxide dismutase activity) [38]. The PC and phosphatidyl ethanolamine (PE) composition in skeletal muscle has been linked to insulin sensitivity. Previous studies have evaluated the relationships between skeletal muscle PC, PE, physical exercise, and insulin sensitivity. Exercise intervention for 12 weeks enhanced insulin sensitivity by 33%, skeletal muscle levels of PC by 21%, PE by 42%, and reduced the PC:PE ratio by 16%. One bicycle session, which is an example of moderate physical activity, reduced PC:PE by 5%. Furthermore, PC:PE correlated negatively with insulin sensitivity and oxidative phosphorylation [39]. Our results showed an over-representation of PC among the active participants, providing further confirmation of the underlying mechanism of physical activity in preventing oxidative stress. All the metabolites mentioned above (plasmalogen, lysoplasmalogen, vitamin A, and PC) can be taken as supplements for their known antioxidant effect. The emerging data have shown that moderate exercise could be used as a strategy to increase their levels without the need for supplement use.

Our results have also shown that glutathione metabolism is one of the enriched pathways associated with moderate physical activity. GSH is a tripeptide thiol antioxidant composed of the amino acids, glutamic acid, cysteine, and glycine [40]. It is synthesized by the sequential addition of cysteine to glutamate, followed by the addition of glycine. The sulfhydryl group (−SH) of cysteine are involved in reduction and conjugation reactions that are usually considered the most important functions of GSH. These reactions provide the means for the removal of peroxides and many xenobiotic compounds. The protective functions of glutathione are mediated through reduction, conjugation, and interaction with other non-enzymatic antioxidants. Most of the GSH in antioxidant defense in cells is utilized by three members of the glutathione peroxidase (GPx) family and by one of the peroxiredoxins (Prdx 6). These enzymes catalyze the reduction of H_2_O_2_ by GSH into H_2_O and GSSG [41].

Since glutathione (GSH) is the most important low molecular weight antioxidant synthesized in cells, we investigated changes in glutathione metabolites in response to moderate exercise in non-obese individuals. Several metabolites related to glutathione metabolism were increased with moderate physical activity, including cysteine-glutathione disulfide, 2-aminobutyrate, cys-gly, oxidized and 2-hydroxybutyrate/2-hydroxyisobutyrate. 2-hydroxybutyrate is related to glutathione synthesis in the sense that, under conditions of oxidative stress, homocysteine metabolism is diverted from the transmethylation pathway (forming methionine) to the transsulfuration pathway (forming cystathionine), and then cysteine, to feed glutathione synthesis. 2-Oxobutyrate, produced along with cysteine from cystathionine, is subsequently reduced to 2-hydroxybutyrate by lactate dehydrogenase. Much like the production of lactate from pyruvate by the same enzyme, it has been reported that an increased [NADH]/[NAD^+^] ratio is the most important factor for the production of 2-hydroxybutyrate. This can explain the exercise-induced increase in 2-hydroxybutyrate, since a highly accelerated glycolytic flux during high-intensity exercise would result in a high rate of NAD^+^-to-NADH conversion at the level of glyceraldehyde 3-phosphate dehydrogenase [42]. Our results also showed elevated cysteine-glutathione disulfide in active individuals, confirming previous studies showing that exhaustive exercise can increase muscle glutathione disulfide content. Concentrations of the glutathione-related amino acids, glutamate, cysteine, and aspartate, were significantly increased in the same muscle after exhaustion [43].

In the present study, glutathione metabolites (Cys-gly, oxidized, 5 oxoproline, cysteinylglycine, and cysteinylglycine disulfide) were associated with higher hemoglobin and hematocrit concentrations in males and females. Recent studies have suggested that a strong effect of oxidative stress (reduced glutathione) on glutathione peroxidase and glutathione reductase levels may reduce hemoglobin concentration in diabetic patients [44]. This in line with our results, where glutathione metabolites (antioxidants) were associated with higher hemoglobin and hematocrit concentrations. When analyzing these associations in males and females separately, our data indicated that the positive associations of Cys-gly, oxidized, and cysteinylglycine with hemoglobin were identified in males, but not in females. This is expected since differences in hemoglobin––among other measured clinical traits, including HDL and sex hormones––are sex-related [45,46].

Our data also revealed that glutathione metabolites, including Cys-gly, oxidized, cysteinylglycine, and cysteinylglycine disulfide, were positively associated with liver function enzymes, including total bilirubin in both genders. Bilirubin can also act as an antioxidant in vitro, but whether its redox activity is physiologically relevant is unclear. Bilirubin’s unique redox activity toward O_2_^⋅−^ may underlie a prominent physiologic role despite being significantly less abundant than other endogenous and exogenous antioxidants [47]. In addition to its antioxidant properties, bilirubin serves as a metabolic hormone that modulates metabolism and immune response. Like other hormones, bilirubin travels through the circulatory system and reaches its target inside cells, primarily PPARα, to elicit gene responses [46,48].

Our data also revealed a positive correlation between uric acid and glutathione metabolites in females. Reduced GSH and uric acid are of particular interest as they are both antioxidants that are closely linked to the energy metabolism homeostasis. The magnitude of increase in plasma uric acid level following a session of intense exercise was previously suggested to be differently affected by the type of exercise performed [49]. Additionally, our data revealed a positive association between the iron profile and glutathione metabolites (Cys-gly, oxidized, cysteinylglycine, and cysteinylglycine disulfide).Previous studies have reported the accumulation of iron, ROS, and glutathione levels, as demonstrated by Wang et al., indicating their intricate relationship in cellular redox regulation and oxidative stress management [50]. 

Our findings are limited by the relatively small sample size and lack of assessment of the physiological mechanisms that are activated by the generation of free radicals during moderate-intensity physical exercise. Future studies will replicate these findings in larger cohorts and will assess the correlation between the identified biomarkers and the previously reported physiological mechanisms that are triggered by free radicals during exercise. Another limitation of our study is the presence of significant differences in multiple related clinical outcomes, such as lipid profile, between moderately active and inactive individuals. While these differences are integral to metabolic processes, considering them as potential confounders might affect the interpretation of our results. It should be noted that while our primary focus is on the correlation analysis across different exercise levels, there is potential value in considering the relationship between oxidative stress markers and clinical traits among sedentary individuals. Such an exploration could provide additional insights into the intricate interplay between oxidative stress and clinical characteristics, which will be considered in future studies.

## 5. Conclusions

This study has shed light on the metabolic pathways underlying the protective effect of moderate exercise in non-obese, apparently healthy individuals, including lysoplasmalogen, plasmalogen, PC, vitamin A, glutathione and related metabolites, and xanthine metabolism. The identification of these metabolites and metabolic pathways in response to moderate physical activity provides a better understanding of exercise physiology as a potent antioxidant mechanism with therapeutic potential. The emerging data offer valuable insights for researchers investigating the long-term effects of regular exercise on the metabolism of healthy, active adults. This is especially significant given that the majority of previous research has focused on short-term responses primarily observed in athletes.

## Figures and Tables

**Table 1:** General characteristics of study participants grouped into active or sedentary.

Clinical Traits	Sedentary (177)	Moderately Active (128)	*p* Value
HOMA-IR (categorical)			
<1.85	87	78	0.048
>1.85	90	50	
Gender			
Male	89	70	0.486
Female	88	58	
Vital signs			
Age	26 (23–28)	26 (24–28)	0.928
BMI	24.31 (22.64–26.68)	24.67 (23.06–27.41)	0.163
Average systolic BP	105 (98–112)	107 (101–115)	0.090
Average diastolic BP	68.6 (7.75)	67.6 (7.45)	0.250
Average pulse rate	69 (63–76)	66.5 (60–73)	0.051
Blood sugar			
Fasting time (minutes)	152 (87–365)	196 (84.5–630.5)	0.138
Glucose (mmol/L)	4.8 (4.6–5.2)	4.9 (4.56–5.2)	0.525
Hb A1c	5.2 (5–5.4)	5.2 (5–5.4)	0.700
Insulin (Uu/mL)	8.2 (6–14.5)	7.5 (5.7–11.825)	0.067
HOMA-IR (continuous)	1.88 (1.325–3.165)	1.6 (1.1425–2.6025)	0.064
C-Peptide ng mL	1.84 (1.415–2.97)	1.74 (1.35–2.51)	0.087
Physical tests			
Sitting height (cm)	91.4 (86.65–134.375)	90.7 (85.5–134.275)	0.407
Weight (kg)	67 (59.2–76.5)	69.95 (61.45–77.325)	0.219
Waist size (cm)	78 (72–87)	79 (73.75–86.25)	0.404
Hip size (cm)	100.8 (6.29)	101 (6.55)	0.804
Waist to hip ratio	0.78 (0.08)	0.79 (0.07)	0.570
Handgrip (left)	28 (21–38)	33 (22–44)	0.015
Handgrip (right)	30 (22–40)	34 (26–48)	0.007
Maximum heart rate (beats/min)	128 (114–141)	120 (109.25–133)	0.002
Run time (seconds)	764 (729–764)	764 (742–764)	0.953
Blood inflammatory markers			
Hemoglobin (g/dL)	13.8 (12.4–15)	13.7 (12.5–15)	0.644
Estimated hemoglobin	17.9 (3.86)	18.6 (3.27)	0.261
Hematocrit %	41.05 (37.9–44.5)	42.1 (38.05–45.05)	0.245
Red blood cell × 10^6^ µL	4.9 (4.5–5.3)	5 (4.5–5.3)	0.438
White blood cell (×10^3^ µL)	6.55 (5.3–7.725)	6.5 (5.5–7.6)	0.711
Monocyte auto %	7.2 (6–8.725)	7.1 (6.2–8.3)	0.571
Monocytes Auto (×10^3^ µL)	0.5 (0.4–0.6)	0.5 (0.4–0.6)	0.779
Neutrophil Auto (×10^3^ µL)	3.5 (2.7–4.5)	3.4 (2.75–4.4)	0.646
Neutrophil auto %	54.4 (9.31)	53.9 (9.91)	0.654
Lymphocyte Auto (×10^3^ µL)	2.2 (1.8–2.6)	2.2 (1.9–2.7)	0.550
Lymphocyte auto %	34.9 (7.69)	35.5 (8.73)	0.562
Eosinophil auto %	2.2 (1.5–3.425)	2.3 (1.35–3.75)	0.685
Eosinophils Auto (×10^3^ µL)	0.1 (0.1–0.2)	0.1 (0.1–0.2)	0.763
Basophil auto %	0.6 (0.4–0.8)	0.6 (0.4–0.7)	0.019
Basophils Auto (×10^3^ µL)	0 (0–0.1)	0 (0–0.01)	0.105
Platelet (×10^3^ µL)	238 (201.75–283.25)	235 (207–269)	0.599
Mean cell hemoglobin (pg)	28.3 (26.175–29.7)	28.2 (26.15–29.5)	0.947
Mean cell hemoglobin concentration (g/dL)	33.4 (32.6–34.025)	33.1 (32.6–33.7)	0.046
C reactive Protein (mg/L)	5 (5–5)	5 (5–5)	0.852
Kidney profile			
Sodium (mmol/L)	140 (139–142)	141 (139–142)	0.142
Potassium (mmol/L)	4.3 (4.1–4.4)	4.3 (4.1–4.5)	0.341
Chloride (mmol/L)	101 (100–102)	101 (100–102)	0.973
Bicarbonate (mmol/L)	26 (25–27)	26 (25–28)	0.212
Urea (mmol/L)	4.2 (3.4–4.8)	4.25 (3.4–5.2)	0.221
Creatinine (mmol/L)	65 (55–77)	68 (54–79)	0.501
Calcium (mmol/L)	2.39 (0.08)	2.4 (0.07)	0.307
Calcium Corrected (mmol/L)	2.27 (0.07)	2.27 (0.06)	0.557
Phosphorus (mmol/L)	1.17 (0.17)	1.19 (0.15)	0.233
Uric Acid (µmol/L)	277 (229–335)	291.5 (238.5–344.25)	0.291
Creatine kinase	78.5 (58–116.5)	91 (66–152)	0.023
Creatine Kinase-1 (ng/mL)	1.16 (0.675–1.745)	1.13 (0.9225–1.495)	0.941
Creatine kinase-2 (u/L)	78 (57.5–122)	82 (59–142.5)	0.631
Magnesium (umol/L)	0.835 (0.05)	0.834 (0.04)	0.813
Homocysteine (umol/L)	8.1 (6.35–9.9)	8.55 (6.6–10.125)	0.383
Total Protein (g/L)	74 (71–76)	74 (72–77)	0.034
Liver profile			
Total bilirubin (umol/L)	7 (5–9)	7 (5–9)	0.963
Albumin (g/L)	46 (45–48)	47 (45–48)	0.531
Alkaline phosphatase (u/L)	65 (56–75)	63 (52–75.25)	0.241
Alanine transaminase (u/L)	17 (13–28)	18 (12–25)	0.955
Aspartate aminotransferase (u/L)	17 (15–21)	18.5 (16–22)	0.030
GGT (u/L)	14 (9–21)	13 (10.25–18.25)	0.547
GGT 2 (u/L)	20 (13–33)	15 (11–22)	0.002
Lipid profile			
HDL Cholesterol (mmol/L)	1.36 (0.33)	1.45 (0.39)	0.031
LDL cholesterol calc (mmol/L)	2.8 (2.2175–3.185)	2.83 (2.0575–3.1025)	0.560
Triglyceride (mmol/L)	0.97 (0.7–1.4)	0.9 (0.6075–1.2)	0.059
Iron profile			
Iron (µmol/L)	15.89 (11–19.97)	15 (10.85–19)	0.513
Total iron binding capacity (µmol/L)	60 (53–65)	58 (54–64.5)	0.348
Unsaturated iron binding capacity (µmol/L)	42 (35–51)	43.15 (36.125–50)	0.995
Ferritin (µg/L)	45 (12–106)	49 (17.75–109.25)	0.395
Hormones			
Free Thyroxine (pmol/L)	13.43 (12.54–14.6)	13.36 (12.48–14.45)	0.589
Free triiodothyronine (nmol/L)	4.54 (4.26–5)	4.5 (4.01–4.88)	0.312
Thyroid stimulating hormone (mU/L)	1.46 (0.95–2.26)	1.305 (0.9975–1.92)	0.223
Total Testosterone (nmol/L)	6.5 (1.36–19.51)	9.115 (1.26–21.69)	0.294
Estradiol (pmol/L)	119.5 (82–265.25)	123.5 (87–236.25)	0.579
Sex hormone binding globulin (nmol/L)	40 (26.4–64.8)	43.05 (28.15–62.62)	0.718
Vitamins			
Folate (nmol/L)	22.7 (8.04)	22.2 (7.66)	0.565
Vitamin B12 (pmol/L)	293.5 (226–375.5)	274.5 (221.25–404.75)	0.717
Dihydroxyvitamin D Total (ng/mL)	14 (11–20)	14 (11–22)	0.788

BMI body mass index, BP blood pressure, LDL low density lipoprotein, HDL high density lipoprotein, HOMA-IR homeostatic model assessment of insulin resistance, Estimated hemoglobin: hemoglobin estimated from hematocrit, Maximum heart rate (beats/min): Estimated calculated maximum heart rate, Waist size (cm): Average waist measurements, Fasting time: Fasting time in minutes. Hip and waist size: Circumference. Data are presented as mean (SD) for normal variables, median (IQR) for skewed variables, and number (percentage) for categorical parameters. Differences between the groups were tested using Student’s *t*-test/Mann–Whitney U test for parametric/non-parametric variables and Fisher’s exact test for nominal variables. A *p*-value significance level of 0.05 was used.

**Table 2:** List of metabolites associated with physical activity status.

Metabolite	Sub-Pathway	Super-Pathway	Estimate	SE	*p*-Value	fdr
10-undecenoate (11:1n1)	Medium chain fatty acid	Lipid	0.22	0.06	7.61 × 10^−5^	0.06
imidazole lactate	Histidine metabolism	Amino Acid	0.12	0.03	2.04 × 10^−4^	0.06
1-palmitoyl-GPA (16:0)	Lysophospholipid	Lipid	−0.24	0.07	2.58 × 10^−4^	0.06
paraxanthine	Xanthine metabolism	Xenobiotics	−0.35	0.10	3.58 × 10^−4^	0.06
undecenoylcarnitine (C11:1)	Fatty acid metabolism (acyl carnitine, monounsaturated)	Lipid	0.21	0.06	3.69 × 10^−4^	0.06
carotene diol (2)	Vitamin A metabolism	Cofactors and Vitamins	0.17	0.05	5.53 × 10^−4^	0.08
thyroxine	Tyrosine metabolism	Amino acid	−0.11	0.03	6.31 × 10^−4^	0.08
fructosyllysine	Lysine metabolism	Amino acid	−0.14	0.04	7.16 × 10^−4^	0.08
carotene diol (1)	Vitamin A metabolism	Cofactors and vitamins	0.16	0.05	1.04 × 10^−3^	0.09
sphingomyelin (d18:2/14:0, d18:1/14:1) *	Sphingomyelins	Lipid	−0.10	0.03	1.16 × 10^−3^	0.09
fructose	Fructose, mannose and galactose metabolism	Carbohydrate	−0.09	0.03	1.23 × 10^−3^	0.09
gamma-glutamyl-2-aminobutyrate	Gamma-glutamyl amino acid	Peptide	0.14	0.04	1.52 × 10^−3^	0.1
N-acetylproline	Urea cycle; arginine and proline metabolism	Amino Acid	−0.13	0.04	1.77 × 10^−3^	0.1
oxalate (ethanedioate)	Ascorbate and aldarate metabolism	Cofactors and Vitamins	0.13	0.04	2.12 × 10^−3^	0.1

Linear regression was used to assess the significance of metabolite associations with active and sedentary physical activity status after correcting for gender, age, BMI, and Principal Components (FDR ≤ 0.1). Asterisks (*) indicated on IDs of some metabolites refer to compounds that have not been officially confirmed based on a standard, but their identities are known with confidence.

**Table 3:** Investigation of glutathione metabolites associated with moderate physical activity.

Glutathione Metabolites	Estimate	SE	*p*-Value
cysteine-glutathione disulfide	0.229	0.078	0.003
2-aminobutyrate	0.089	0.034	0.009
cys-gly, oxidized	0.091	0.039	0.020
2-hydroxybutyrate/2-hydroxyisobutyrate	0.094	0.050	0.051
cysteinylglycine disulfide *	0.026	0.025	0.296
cysteinylglycine	−0.011	0.054	0.836
5-oxoproline	0.001	0.020	0.962

Asterisks (*) indicated on IDs of some metabolites refer to compounds that have not been officially confirmed based on a standard, but their identities are known with confidence.

## Data Availability

Data is contained within the article.

## Appendix A

**Table A1:** Demographics of participants categorized by gender and physical activity.

	Males			Females		
Clinical Traits	Sedentary (89)	Moderately Active (70)		Sedentary (88)	Moderately Active (58)	
HOMA-IR (categorical)						
<1.85	56	44	0.993	54	34	0.740
>1.85	33	26		34	24	
Vital signs						
Age	26 (24–28)	26 (24–28)	0.807	26 (23–28)	25 (23–28)	0.586
BMI	25.33 (23.05–27.28)	25.5 (23.65–27.39)	0.381	23.8 (22.25–26.145)	24.235 (21.99–27.41)	0.507
Average systolic BP	112 (106–119)	113 (106–119)	0.718	98 (93.75–104)	101 (97–106)	0.031
Average diastolic BP	71 (65–76)	69 (62.5–75)	0.167	66 (62–70)	66.5 (61.25–71)	0.911
Average pulse rate	66 (61–73)	64 (59–71.75)	0.258	71 (67–77.25)	70 (63.25–76)	0.165
Blood sugar						
Fasting time (minutes)	139 (74–259)	209 (100–635	0.027	168 (94–569.75)	174 (76.5–593.25)	0.940
Glucose (mmol/L)	5 (4.7–5.4)	4.9 (4.7–5.2)	0.213	4.7 (4.5–5)	4.8 (4.5–5.065)	0.994
Hb A1c	5.2 (5.1–5.4)	5.3 (5.1–5.4)	0.638	5.2 (5–5.4)	5.2 (5–5.4)	0.335
Insulin (Uu/mL)	10 (6.4–16.775)	7 (5.47–11.87)	0.015	7.5 (6–10.15)	8 (6–11)	0.903
HOMA-IR (continuous)	2.195 (1.39–3.735)	1.585 (1.11–2.73)	0.011	1.59 (1.215–2.28)	1.76 (1.195–2.43)	0.936
C-Peptide ng mL	2.13 (1.57–3.39)	1.78 (1.34–2.79)	0.043	1.65 (1.39–2.42)	1.7 (1.36–2.495)	0.723
Physical tests						
Sitting height (cm)	91.4 (88.3–96.5)	91.95 (88.27–136.12)	0.898	89.2 (84–134.65)	85.6 (82.875–133.575)	0.163
Weight (kg)	75.7 (68.6–82.7)	76.3 (69.175–83.625)	0.537	60.6 (57.7–66.225)	61.3 (56.55–70.825)	0.722
Waist size (cm)	87 (79–90)	83.5 (79–89)	0.416	73 (69–77)	74 (69–79)	0.319
Hip size (cm)	99 (96–104)	101 (96–105)	0.491	101 (97–106)	103 (97–107)	0.805
Waist to hip ratio	0.847 (0.8–0.89)	0.84 (0.795–0.865)	0.211	0.725 (0.68–0.75)	0.72 (0.70–0.77)	0.331
Handgrip (left)	38 (34–44)	43.5 (36.25–48)	0.007	22 (18–24)	22 (18–28)	0.200
Handgrip (right)	40 (36–46)	46.5 (39.25–50)	0.002	22.5 (20–28)	26 (20.25–28)	0.092
Maximum heart rate (beats/min)	118.5 (108–128)	114 (106–125)	0.292	139 (129–150)	131 (120–139)	0.001
Run time (seconds)	764 (742–764)	764 (742–764)	0.795	743 (684–764)	743 (684–764)	0.912
Blood inflammatory markers						
Hemoglobin (g/dL)	15 (14.4–15.7)	14.9 (14.5–15.6)	0.616	12.4 (11.15–13.25)	12.45 (11.8–13)	0.516
Estimated hemoglobin *	19.65 (18.22–20.295)	18.9 (17.65–20.54)	0.394	16. 7 (3.8)	16.98 (15.36–21.02)	0.078
Hematocrit %	44.5 (42.7–46.5)	44.7 (43–46.3)	0.413	37.9 (34.85–39.85)	37.65 (36.025–39.55)	0.356
Red Blood Cell × 10^6^ µL	5.2 (5–5.6)	5.3 (5–5.5)	0.898	4.5 (4.3–4.8)	4.5 (4.225–4.9)	0.780
White Blood Cell (×10^3^ µL)	5.8 (4.9–7)	6.3 (5.5–7.4)	0.128	6.8 (5.75–7.95)	6.75 (5.425–7.9)	0.430
Monocyte auto %	8.1 (6.8–9.5)	7.6 (6.4–8.8)	0.129	6.4 (5.6–7.95)	7 (5.525–7.675)	0.678
Monocytes Auto (×10^3^ µL)	0.5 (0.4–0.6)	0.5 (0.4–0.6)	0.981	0.4 (0.4–0.6)	0.4 (0.4–0.5)	0.661
Neutrophil Auto (×10^3^ µL)	3 (2.5–3.9)	3.3 (2.7–4.1)	0.193	4 (3.15–4.9)	3.4 (2.825–4.4)	0.084
Neutrophil auto %	51.3 (46.2–58.5)	53.4 (9.64)	0.347	57.2 (8.42)	54.5 (10.26)	0.034
Lymphocyte Auto (×10^3^ µL)	2.2 (1.7–2.6)	2.2 (1.9–2.4)	0.936	2.2 (1.85–2.65)	2.3 (1.925–2.8)	0.366
Lymphocyte auto %	36.4 (30.7–40.7)	34.8 (31.3–40.8)	0.276	32.5 (28.6–36.7)	35.85 (31.1–42.475)	0.016
Eosinophil auto %	2.9 (1.7–3.6)	2.9 (1.6–4.3)	0.671	1.7 (1–2.7)	1.8 (1.125–2.85)	0.601
Eosinophils Auto (×10^3^ µL)	0.2 (0.1–0.2)	0.2 (0.1–0.3)	0.793	0.1 (0.1–0.2)	0.1 (0.1–0.2)	0.825
Basophil auto %	0.6 (0.4–0.8)	0.6 (0.4–0.7)	0.318	0.7 (0.45–0.8)	0.5 (0.3–0.8)	0.023
Basophils Auto (×10 µL)	0 (0–0.1)	0 (0–0)	0.317	0 (0–0.1)	0 (0–0.0325)	0.216
Platelet (×10^3^ µL)	211 (180–259.75)	220 (197–254)	0.356	268.5 (233–302.75)	254.5 (218.5–297.75)	0.308
Mean Cell Hemoglobin (pg)	28.9 (27.2–29.9)	28.5 (26.7–29.9)	0.813	27.8 (24.8–29.45)	27.65 (25.725–29.2)	0.974
Mean Cell Hemoglobin Concentration (g/dL)	33.8 (33.2–34.5)	33.5 (32.9–34)	0.007	33 (32.2–33.6)	32.8 (32.325–33.475)	0.546
C Reactive Protein (mg/L)	5 (5–5)	5 (5–5)	0.900	5 (5–5)	5 (5–5)	0.990
Kidney profile						
Sodium (mmol/L)	141 (140–142)	141 (140–142)	0.678	139 (138–141)	140 (139–141)	0.098
Potassium (mmol/L)	4.3 (4.1–4.4)	4.3 (4.2–4.5)	0.058	4.25 (4–4.425)	4.2 (4–4.4)	0.594
Chloride (mmol/L)	101 (100–102)	101 (99.25–102)	0.757	101 (100–103)	101 (100–102)	0.897
Bicarbonate (mmol/L)	27 (26–28)	27.5 (26–28)	0.106	26 (24–27)	25 (24–27)	0.775
Urea (mmol/L)	4.6 (4–5.2)	4.8 (3.95–5.4)	0.424	3.6 (2.975–4.3)	3.7 (3–4.875)	0.655
Creatinine (mmol/L)	77 (71–83)	77.5 (72–85.5)	0.238	55 (51–61)	54 (49.5–60)	0.424
Calcium (mmol/L)	2.42 (0.07)	2.41 (0.06)	0.730	2.37 (0.07)	2.39 (0.07)	0.113
Calcium Corrected (mmol/L)	2.27 (0.07)	2.27 (0.07)	0.932	2.27 (0.06)	2.27 (0.06)	0.441
Phosphorus (mmol/L)	1.15 (1.18)	1.18 (0.17)	0.319	1.18 (0.16)	1.20 (0.13)	0.447
Uric Acid (µmol/L)	333 (301–369)	338.5 (308.25–380.75)	0.714	230 (199–254.5)	238.5 (202–267)	0.459
Creatine kinase	113 (78–171.5)	121 (95.5–255)	0.036	60 (48.5–81.5)	66.5 (53.25–85.75)	0.291
Creatine Kinase_1 (ng/mL)	1.65 (1.1025–1.865)	1.385 (1.125–1.6625)	0.725	1.02 (0.6075–1.395)	0.89 (0.525–1.1425)	0.577
Creatine kinase_2 (u/L)	112 (72.75–155.5)	161 (115–255)	0.151	58 (53.5–80.5)	67 (51.25–83.5)	0.805
Magnesium (µmol/L)	0.835 (0.05)	0.83 (0.04)	0.655	0.836 (0.05)	0.82 (0.05)	0.451
Homocysteine (µmol/L)	8.95 (7.78–11.025)	9.1 (7.55–10.75)	0.689	7.5 (5.9–8.95)	7.55 (6.075–9.7)	0.546
Total Protein (g/L)	74 (71–76)	74 (71–76)	0.442	73 (70–75.25)	74.5 (72–77)	0.024
Liver profile						
Total bilirubin (µmol/L)	8 (5.85–10)	7.5 (5.7–12)	0.532	6.05 (4.075–8)	5.7 (4.2–7.4)	0.344
Albumin (g/L)	47 (46–49)	47 (46–49)	0.689	45 (44–47)	46 (45–47)	0.131
Alkaline phosphatase (u/L)	70 (60–79)	64.5 (55.25–77.5)	0.061	59.5 (51–69)	59.5 (48.75–71.75)	0.916
Alanine transaminase (u/L)	24 (18–38)	22 (17–34)	0.419	13 (9.75–17)	12.5 (11–16.75)	0.744
Aspartate aminotransferase (u/L)	19 (16–23)	21 (17–26)	0.065	16 (13.75–19)	17 (14.25–19)	0.169
GGT (u/L)	20.5 (17–28.75)	16 (12.5–23.5)	0.025	10 (8–14)	11 (9–13.25)	0.951
GGT 2 (u/L)	24 (16–38)	17 (13–23)	0.002	14 (11.75–18.5)	11 (9–17.75)	0.051
Lipid profile						
HDL Cholesterol (mmol/L)	1.20 (0.31)	1.29 (0.34)	0.100	1.53 (0.26)	1.66 (0.35)	0.018
LDL Cholesterol Calc (mmol/L)	2.96 (2.475–3.715)	2.85 (2.085–3.42)	0.127	2.635 (2.06–3)	2.8 (2.01–3.03)	0.518
Triglyceride (mmol/L)	1.12 (0.86–1.5)	0.98 (0.6225–1.2975)	0.010	0.8 (0.55–1.10)	0.8 (0.6025–1)	0.974
Iron profile						
Iron (umol/L)	18 (14.91–21.87)	16.595 (14.08–20.61)	0.286	12.95 (7.205–17.075)	12 (8.79–15)	0.995
TIBC (umol/L)	55 (51–59.5)	55 (52.25–60)	0.491	64 (60–73.5)	61 (57–70)	0.083
UIBC (umol/L)	37.25 (32.225–42)	39 (31.6–45.2)	0.222	50.7 (43–63.5)	48.9 (42–61.2)	0.382
Ferritin (µg/L)	101 (62.5–149)	105 (57.25–133.75)	0.653	13.5 (7–28.25)	19 (9–33.75)	0.200
Hormones						
Free Thyroxine (pmol/L)	13.4 (12.61–14.58)	13.36 (12.49–14.455)	0.449	13.47 (12.3075–14.7)	13.35 (12.395–14.44)	0.994
Free triiodothyronine (nmol/L)	4.7 (4.4–5.1)	4.6 (4.34–4.9475)	0.146	4.38 (4–4.85)	4.4 (3.95–4.8)	0.733
TSH (mU/L)	1.43 (0.95–2.14)	1.22 (0.9925–1.8275)	0.263	1.535 (0.915–2.46)	1.34 (1.035–1.92)	0.537
Total Testosterone (nmol/L)	19.51 (15.56–23.06)	21.34 (16.99–26.64)	0.135	1.335 (0.935–1.61)	1.19 (0.9–1.49)	0.583
Estradiol (pmol/L)	86 (69.25–113)	98 (77–121)	0.107	285 (148.75–596)	246 (165–505)	0.848
SHBG (nmol/L)	28.65 (22.15–38.2)	33 (24–43.1)	0.180	61 (45–102.7)	64 (45–91)	0.887
Vitamins						
Folate (nmol/L)	20.49 (8.0)	21.05 (16.325–26.175)	0.332	25.1 (7.74)	23.2 (17.1–28.7)	0.235
Vitamin B12 (pmol/L)	331.5 (245.25–422.75)	322 (249–429.5)	0.927	270.5 (216.5–327.5)	252 (200.5–309)	0.237
Dihydroxyvitamin D Total (ng/mL)	14 (12–20)	14 (12–21.75)	0.927	14 (10–20.5)	14.5 (10.25–23)	0.743

BMI body mass index, BP blood pressure, LDL low density lipoprotein, HDL high density lipoprotein, HOMA-IR homeostatic model assessment of insulin resistance, * hemoglobin estimated from hematocrit. Data are presented as mean (SD) for normal variables, median (IQR) for skewed variables, and number (percentage) for categorical parameters. Differences between the groups were tested using Student’s *t*-test/Mann–Whitney U test for parametric/non-parametric variables and Fisher’s exact test for nominal variables. A *p*-value significance level of 0.05 was used.

**Table A2:** Significant metabolites from the linear regression analysis that belong to enriched pathways.

Metabolite	Sub-Pathway	Super-Pathway	Estimate	SE	*p*-Value
cysteine-glutathione disulfide	Glutathione Metabolism	Amino Acid	0.23	0.08	0.003
2-aminobutyrate	Glutathione Metabolism	Amino Acid	0.09	0.03	0.009
cys-gly, oxidized	Glutathione Metabolism	Amino Acid	0.09	0.04	0.02
1-(1-enyl-oleoyl)-GPE (P-18:1) *	Lysoplasmalogen	Lipid	0.12	0.04	0.003
1-(1-enyl-stearoyl)-GPE (P-18:0) *	Lysoplasmalogen	Lipid	0.11	0.04	0.007
1-(1-enyl-palmitoyl)-GPC (P-16:0) *	Lysoplasmalogen	Lipid	0.08	0.03	0.01
1-(1-enyl-palmitoyl)-GPE (P-16:0) *	Lysoplasmalogen	Lipid	0.09	0.04	0.019
1-myristoyl-2-arachidonoyl-GPC (14:0/20:4) *	Phosphatidylcholine (PC)	Lipid	−0.16	0.06	0.011
1-palmitoyl-2-dihomo-linolenoyl-GPC (16:0/20:3n3 or 6) *	Phosphatidylcholine (PC)	Lipid	−0.11	0.04	0.013
1-myristoyl-2-palmitoyl-GPC (14:0/16:0)	Phosphatidylcholine (PC)	Lipid	−0.15	0.06	0.018
1-palmitoyl-2-palmitoleoyl-GPC (16:0/16:1) *	Phosphatidylcholine (PC)	Lipid	−0.11	0.05	0.021
1-stearoyl-2-linoleoyl-GPC (18:0/18:2) *	Phosphatidylcholine (PC)	Lipid	−0.05	0.02	0.038
1-stearoyl-2-oleoyl-GPC (18:0/18:1)	Phosphatidylcholine (PC)	Lipid	−0.07	0.03	0.048
1-(1-enyl-palmitoyl)-2-arachidonoyl-GPC (P-16:0/20:4) *	Plasmalogen	Lipid	0.08	0.03	0.009
1-(1-enyl-palmitoyl)-2-oleoyl-GPC (P-16:0/18:1) *	Plasmalogen	Lipid	0.08	0.03	0.013
1-(1-enyl-palmitoyl)-2-linoleoyl-GPC (P-16:0/18:2) *	Plasmalogen	Lipid	0.08	0.03	0.018
1-(1-enyl-stearoyl)-2-arachidonoyl-GPE (P-18:0/20:4) *	Plasmalogen	Lipid	0.09	0.04	0.02
1-(1-enyl-palmitoyl)-2-arachidonoyl-GPE (P-16:0/20:4) *	Plasmalogen	Lipid	0.08	0.04	0.02
1-(1-enyl-palmitoyl)-2-oleoyl-GPE (P-16:0/18:1) *	Plasmalogen	Lipid	0.07	0.03	0.026
carotene diol (2)	Vitamin A Metabolism	Cofactors and Vitamins	0.17	0.05	0.001
carotene diol (1)	Vitamin A Metabolism	Cofactors and Vitamins	0.16	0.05	0.001
carotene diol (3)	Vitamin A Metabolism	Cofactors and Vitamins	0.11	0.05	0.043
paraxanthine	Xanthine Metabolism	Xenobiotics	−0.35	0.1	<0.001
theophylline	Xanthine Metabolism	Xenobiotics	−0.25	0.09	0.009
1-methylurate	Xanthine Metabolism	Xenobiotics	−0.2	0.1	0.035
caffeine	Xanthine Metabolism	Xenobiotics	−0.29	0.14	0.044

Asterisks (*) indicated on IDs of some metabolites refer to compounds that have not been officially confirmed based on a standard, but their identities are known with confidence.
